# The Effectiveness of Lower-Limb Wearable Technology for Improving Activity and Participation in Adult Stroke Survivors: A Systematic Review

**DOI:** 10.2196/jmir.5891

**Published:** 2016-10-07

**Authors:** Lauren Powell, Jack Parker, Marrissa Martyn St-James, Susan Mawson

**Affiliations:** ^1^ School of Health and Related Research (ScHARR) University of Sheffield Sheffield United Kingdom

**Keywords:** wearable technology, stroke, gait, rehabilitation

## Abstract

**Background:**

With advances in technology, the adoption of wearable devices has become a viable adjunct in poststroke rehabilitation. Regaining ambulation is a top priority for an increasing number of stroke survivors. However, despite an increase in research exploring these devices for lower limb rehabilitation, little is known of the effectiveness.

**Objective:**

This review aims to assess the effectiveness of lower limb wearable technology for improving activity and participation in adult stroke survivors.

**Methods:**

Randomized controlled trials (RCTs) of lower limb wearable technology for poststroke rehabilitation were included. Primary outcome measures were validated measures of activity and participation as defined by the International Classification of Functioning, Disability and Health. Databases searched were MEDLINE, Web of Science (Core collection), CINAHL, and the Cochrane Library. The Cochrane Risk of Bias Tool was used to assess the methodological quality of the RCTs.

**Results:**

In the review, we included 11 RCTs with collectively 550 participants at baseline and 474 participants at final follow-up including control groups and participants post stroke. Participants' stroke type and severity varied. Only one study found significant between-group differences for systems functioning and activity. Across the included RCTs, the lowest number of participants was 12 and the highest was 151 with a mean of 49 participants. The lowest number of participants to drop out of an RCT was zero in two of the studies and 19 in one study. Significant between-group differences were found across three of the 11 included trials. Out of the activity and participation measures alone, *P* values ranged from *P*=.87 to *P* ≤.001.

**Conclusions:**

This review has highlighted a number of reasons for insignificant findings in this area including low sample sizes, appropriateness of the RCT methodology for complex interventions, a lack of appropriate analysis of outcome data, and participant stroke severity.

## Introduction

The worldwide incidence of stroke is set to escalate from 15.3 million to 23 million by 2030 [1]. In the United Kingdom, strokes are the largest single cause of disability [2] resulting in a cost to the economy of £8.9 billion a year [3]. It is estimated that following a stroke, only 15% will gain complete functional recovery for both the upper and lower extremities [4] with walking and mobility being key issues for many stroke survivors who report the importance of regaining mobility [5]. However, with the ever-increasing financial challenges facing the National Health Service (NHS), service needs cannot be met. Therefore, utilizing information and communication technology together with the implementation of well-evidenced medical technologies is essential for continued rehabilitation for stroke survivors.

The adoption of technological solutions can facilitate patient and caregiver empowerment and a paradigm shift in control and decision making to that of a shared responsibility and self-management [6]. It also has the potential to reduce the administrative burden for care professionals and support the development of new interventions [7]. Incorporating technology into the daily lives of stroke survivors is a key objective in safeguarding a better quality of life for them.

Evidence exists supporting the need for intensity and repetition of motor skills in order to promote neuroplasticity and motor relearning [8]. A number of technological aids with a potential to enhance poststroke motor recovery has been explored [9]. However, many include the use of expensive, large, complex, cumbersome apparatus that necessitates the therapist to be present during use [10]. Therefore inexpensive, externally wearable, commercially available sensors have become a more viable option for independent home-based poststroke rehabilitation [11].

Recent systematic and non-systematic reviews highlight the growing use of externally wearable devices to augment poststroke rehabilitation in both clinical and non-clinical settings for motion analysis and physical activity monitoring [12-15]. These include microelectromechanical systems containing accelerometers, gyroscopes, and magnetometers; fabric and body-worn sensor networks [16]; and physiological monitoring such as blood pressure and oxygen saturation [17,18]. Other wearable devices specifically designed and used for poststroke rehabilitation also include robotics [19], virtual reality [20], Functional Electrical Stimulation (FES) [21], electromyographic biofeedback (EMG-BFB) [22], and Transcutaneous Electrical Nerve Stimulation (TENS) [23,24].

However, while these devices have the potential to reliably measure duration, frequency, intensity, and quality of activity and movement, all of which are key variables for poststroke recovery [8], no reviews have synthesized the effectiveness of these devices for poststroke lower-limb rehabilitation.

The International Classification of Functioning, Disability and Health (ICF) [25] considers the interaction between pathology (body structure and function), impairment (signs and symptoms), activities (functionality), and participation (social integration) and has now become the main conceptual framework for poststroke rehabilitation [26-28]. For this review, we focused on the activities and participation domain of the ICF as this would provide an indication of how the interventions have or have not led to functional gains in everyday life, which is the rehabilitation goal for both clinicians and stroke survivors [28].

Therefore, the aim of this review was to examine how effective external wearable devices are as interventions for improving function of the lower limb in adult stroke survivors.

## Methods

The review protocol was registered on PROSPERO (CRD42015020544). The review was undertaken in accordance with the general principles recommended in the Preferred Reporting Items for Systematic Reviews and Meta-Analyses (PRISMA) [29].

### Search Methods

The following databases were searched from inception to March 2016: MEDLINE, Web of Science (Core collection), CINAHL, and the Cochrane Library. Medical Subject Headings (MeSH) keywords used were cerebrovascular disorders, hemorrhage, cerebral hemorrhage, self-help devices, telemedicine, physical therapy modalities, physical and rehabilitation medicine, exercise, exercise therapy, exercise movement techniques, self-evaluation programs, sensory feedback, motor skills, gait disorders, neurologic, gait apraxia, and gait ataxia. Text terms used were stroke, technology, physiotherapy, lower limb, rehabilitation, and gait. These were combined with text term synonyms: cerebrovascular accident (CVA), poststroke, cerebrovascular, brain ischemia, IT (information technology), ICT (information and communications technology), assistive technology, telehealth, telecare, telerehabilitation, physical therapy, physiatric, exercise, lower extremity, lower limb, ambulant, walk, locomotion, mobile, move, motion, biofeedback, sensory feedback, advise, result, evaluation, observe, assess, inform, train, therapy, treat, motor skills, motor re-learn, re-educate, re-learn, recovery enhance, promote, support, function, activity, physical, ambulant, and walking. Terms were combined using Boolean logic (“AND”, “OR”). MeSH are specific recognized terms used for the purpose of indexing journal articles and books in electronic databases. Free text terms and synonyms are specific words that the search strategy looks for in the title and abstract.

A copy of the MEDLINE search strategy is presented in Multimedia Appendix 1. Electronic citations were downloaded to Endnote software. The inclusion criteria are described in Table 1.

**Table 1:** Inclusion and exclusion criteria for this review.

Inclusion criteria	Exclusion criteria
English language articles	Studies including upper limb
Studies recruiting people over the age of 18 years	Studies where the intervention is not clearly defined
Studies evaluating lower-limb and wearable technology	Studies not using one of the chosen 11 outcome measures (see Outcome measurement/assessment below)
Studies reporting an RCT^a^	Studies not reporting an RCT^a^
Studies measuring activity and participation as classified by the World Health Organization ICF^b^	Studies not measuring activity and participation as classified by the World Health Organization ICF^b^

^a^RCT: randomized controlled trial.

^b^ICF: International Classification of Functioning, Disability and Health.

As this is a review of effectiveness, RCTs were chosen as the appropriate study design to answer the research question. Inclusion of non-RCT evidence is outside the scope of this review.

Comparators could be exercise/physical therapy, sham stimulation, conventional gait therapy, or treatment as usual. The primary outcome for this review was changes in activity and participation assessed by any of the following methods: the Rivermead Mobility Index, the Barthel Index, the Berg Balance Scale, the Six Minute Walk Test, the Functional Ambulatory Category, the Timed Up and Go test, the Motricity Index, the Stroke Self-Efficacy Scale, and the Performance-Oriented Mobility Assessment.

### Quality Assessment

Methodological quality of included RCTs was assessed using the Cochrane Collaboration risk of bias assessment criteria [30]. This tool addresses specific domains, namely, sequence generation, allocation concealment, blinding of participants and personnel, blinding of outcome assessment, incomplete outcome data, and selective outcome reporting. For the selective reporting domain, a proxy judgement was made that if a trial reported that a study protocol had been approved and the trial report described primary and secondary outcomes with results, then the trial could be considered at low risk of selective reporting bias. We classified RCTs as being at overall low risk of bias if they were rated as “low” for each of three key domains: (1) allocation concealment [31], (2) blinding of outcome assessment, and (3) completeness of outcome data. RCTs judged as being at high risk of bias for any of these domains were judged at overall high risk. Similarly, RCTs judged as being at unclear risk of bias for any of these domains were judged at overall unclear risk.

### Data Extraction

Retrieved titles, abstracts, and/or papers were screened independently by 2 review authors (LAP, JP) to identify studies that met the inclusion criteria. Disagreements were resolved between reviewers through discussion. A standardized form was used for data extraction using Excel. Details of the RCT characteristics, included participants, the intervention, and comparator. Data extraction was carried out by reviewer LP and checked for accuracy by reviewer JP. Missing data were requested from study authors.

### Outcome Measurement Assessment

When undertaking a systematic review, it is essential that the quality of the outcome measures used in each study is assessed in order to ensure that the results of the study are valid and reliable. In order to do this, three clear domains need to be considered for each of the outcome measures used: (1) whether the psychometric properties of the scale have been assessed previously [32], (2) whether the clinimetric properties of the scale have been considered [33-37], specifically the Minimally Clinically Important Difference (MCID) [36], and (3) whether the design and analysis of the measurement scale fulfils the requirements of measurement theory [38-40].

We identified all the outcome measures (N=19) used in the 11 trials and reviewed each individually to assess whether they fulfilled the first two domains outlined above. The outcome measures were:

- The Rivermead Mobility Index (RMI)
- 10 Meter Walk Test (10MWT)
- Nottingham Activities of Daily Living Index (ADL)
- The Barthel Index (BI)
- The Berg Balance Scale (BBS)
- 6 Minute Walk Test (6MWT)
- Functional Ambulatory Category (FAC)
- Timed Up and Go Test (TUG)
- Emory Functional Ambulation Profile (EFAP)
- Short Physical Performance Battery (SPPB)
- Performance-Oriented Mobility Assessment (POMA)
- Motricity Index (MI)
- Average Daily Walking Time
- Fastest Safe 15-meter Walking Speed
- Changes in Walking Duration
- Step Numbers
- Daily Walking Activities with an average cadence of walking events (bouts)
- Stroke Impact Scale (SIS)
- Stroke Self-Efficacy Questionnaire (SEQ)

This was established by reviewing the literature on each of the measuring scales. We then examined each measurement scale to establish how the data were scored and how data collected were subsequently analyzed within the results section of each trial.

We classified the measures against the three domains within the World Health Organization ICF, as the aim of this review was to assess the effectiveness of lower-limb wearable technology for improving activity and participation. We wanted to exclude any measurements of “body structures” (impairment) such as the Fugl-Meyer assessment or the Ashworth scale. All 19 outcome measures included were measures of “activity” and 2 were measure of “participation” as classified by the ICF [23].

### Data Synthesis

We have presented a narrative overview of the included RCTs with supporting evidence tables and text. A meta-analysis was not undertaken.

## Results

### Search Results

**Figure 1:**
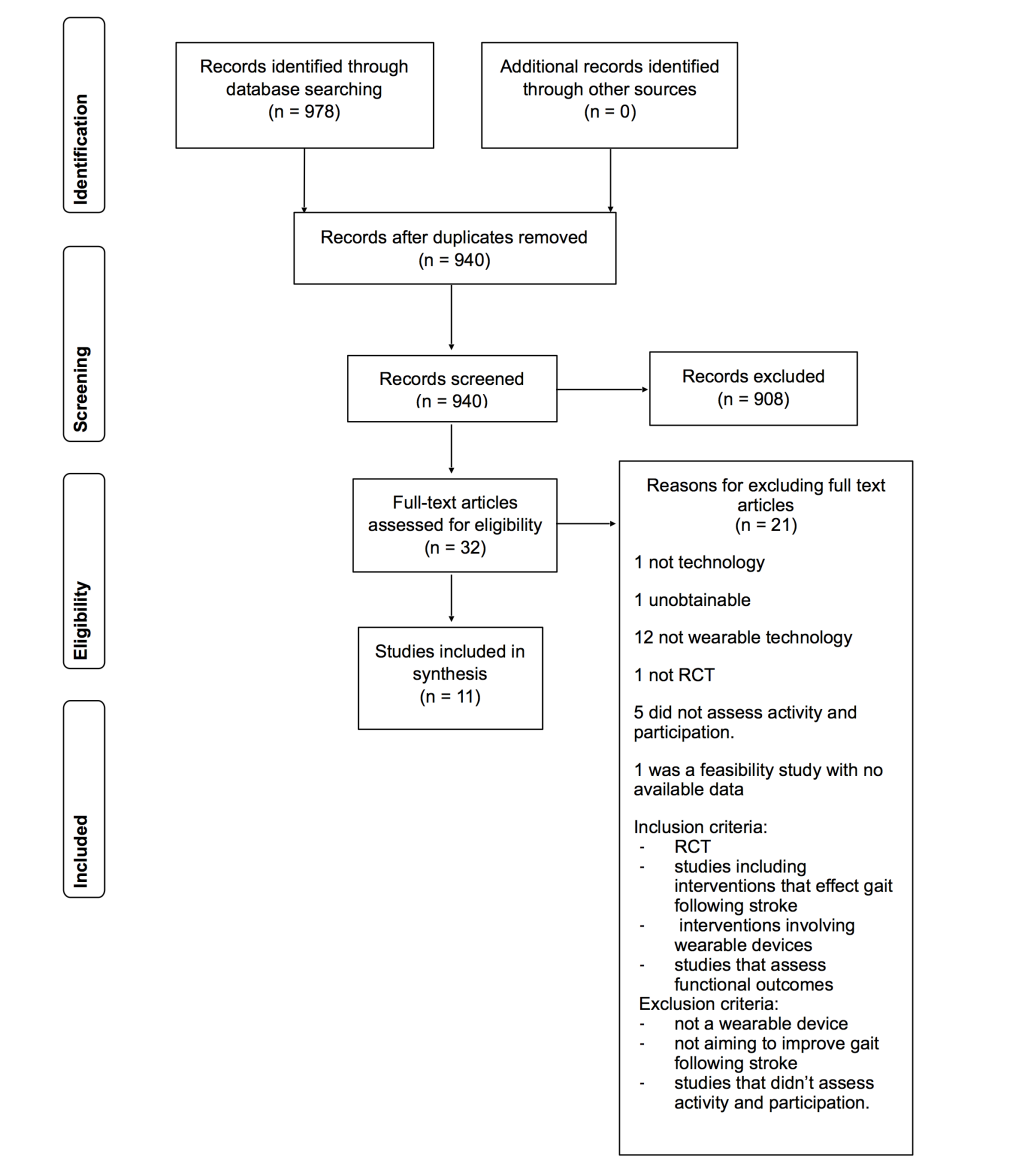
Selection of articles for review.

The electronic searches identified 940 citations following de-duplication. No additional citations were identified through reference searches/other sources. We excluded 780 citations at the title and 128 at abstract stage. We then obtained 32 citations as full-text articles. Of these, 21 were excluded at the full-text stage; details of these excluded studies with the reason for exclusion are shown in Multimedia Appendix 2 [41-59]. Eleven RCTs reported across 11 publications were included in the review (see Figure 1).

### Quality Assessment

Full details from the Cochrane risk of bias assessment are presented in Multimedia Appendix 3. A summary of the risk of bias assessment is presented in Table 2, and a summary of the outcome measurement quality assessment can be found in Multimedia Appendix 4.

Seven of the 11 included RCTs were considered to be at overall high risk of bias [60-66]. Six of these were judged to be at high risk of an attrition bias [60-63,65,66], and two reported that the outcome assessment was not blinded [64,66]. The remaining three RCTs were considered to be at overall unclear risk of bias. None of the included RCTs were considered to be at high risk for the concealment of allocation domain.

**Table 2:** Risk of bias summary.

	Random sequence generation	Allocation concealment	Blinding of participants and personnel	Blinding of outcome assessment	Incomplete outcome data	Selective reporting	Overall
Bauer, 2015 [60]	Low risk	Low risk	High risk	Low risk	High risk	Low risk	High risk
Bradley, 1998 [61]	Unclear	Unclear	Unclear	Unclear	High risk	Unclear	High risk
Dorsch, 2015 [62]	Low risk	Unclear	Unclear	Low risk	High risk	Low risk	High risk
Intiso, 1994 [63]	Unclear	Unclear	Unclear	Low risk	High risk	Unclear	High risk
Mansfield, 2015 [67]	Low risk	Low risk	Unclear	Low risk	Low risk	Low risk	Low risk
Mirelman, 2009 [68]	Unclear	Unclear	High risk	Unclear	Low risk	Unclear	Unclear
Salisbury, 2013 [64]	Low risk	Low risk	Unclear	High risk	Low risk	Unclear	High risk
Shamay, 2009 [65]	Low risk	Unclear	High risk	Low risk	High risk	Unclear	High risk
Solopova, 2011 [69]	Unclear	Unclear	Unclear	Unclear	Low risk	Unclear	Unclear
Stein, 2014 [14]	Unclear	Unclear	Unclear	Unclear	Low risk	Low risk	Unclear
Watanabe, 2014 [66]	High risk	Unclear	High risk	High risk	High risk	Low risk	High risk

### Quality Assessment of Measurement Scales

Eight of the 11 [14,60-62,64,66-68] included RCTs used a combination of ordinal and ratio scales of measurement all with established psychometric properties; however, it was unclear what the minimally clinically important difference (MCID) was for the ratio data. Therefore, the clinical significance of the findings is difficult to establish. Two papers [63,69] used the Bartell Index alone, which has been proven not to be a unidimensional scale. Therefore, the analysis of the data was inappropriate, putting the findings at risk. One paper [65] used ratio levels of measurement, but again the MCID was unclear. Relative results are not reported in the RCTs.

## Discussion

### Principal Findings

This review set out to answer the question “What is the effectiveness of lower-limb wearable technology for improving activity and participation in adult stroke survivors?” The review found that there is little evidence in the literature to support the use of wearable technologies to improve activity and participation. Following exclusions, outcome measure assessment and quality assessment of RCTs, 11 studies were included (see Table 3).

The interventions used in eight of the 11 RCTs identified made no significant between-group differences in functional and participation abilities in adults post stroke. Three of the 11 studies did demonstrate significant between-group differences. One study that found significant between-group differences recruited 109 participants [65] comparing TENs together with a task-related exercise program modified from the training programs [70] with placebo and exercise and a control with no active treatment. The study provided evidence that the subjects receiving this intervention in a home environment had a significantly greater absolute and percentage increase in gait velocity and a reduction in timed get up and go scores from Week 2 onwards.

Another study recruited 60 participants [67] where all participants wore accelerometers around both ankles and were randomly assigned to either receive feedback on the accelerometer data from their physiotherapist or to not receive feedback. The study provided evidence that providing feedback to the participants significantly improved their cadence of daily walking.

The third study recruited 40 participants [60] where all participants underwent 20 minutes of active leg cycling with or without FES application to the muscles of the paretic upper leg. The study provided evidence for the intervention improving participants gait and balance (measured using the POMA); however, these improvements were not sustained when participants were followed up. It could be argued, however, that the high dropout rate (n=19) could have affected the significance of the lasting effects of the study.

As described fully in the quality assessment section of this paper, seven of the 11 included RCTs were considered to be at overall high risk of bias [66]. However, this does not mean that the interventions were not effective for improving gait for people post stroke. A number of conclusions could be drawn from this result. One may be that interventions that rely heavily on direct clinical input may not be suitable for this population where self-managed interventions may be more appropriate.

A number of measurement scales used in the trials were not incorporated in the outcome data for the review, as they were not validated scales: the Bobath scale [61], the 5X Sit-To-Stand-Test [14], and the California Functional Evaluation 40 [14]. Of the 11 RCTs included in the review, eight used a combination of ordinal and ratio data with proven psychometric properties; however, the clinicmetric properties were not described. The lack of evidence, therefore, in eight studies could have been due to the lack of a clinically meaningful, responsive outcome measurement scale combined with a potential lack of statistical power due to small sample sizes. The three studies that did have significant results used a combination of ordinal and ratio data with only one study [60] that provided estimates of MCID together with appropriate anayisis of the FAC data. While Shamay et al did not consider the clinical meaning or significance of the change in scores, they did report research supporting the “practical significance” of the TUG [71], which found that older adults who were able to complete the TUG task in less than 20 seconds were more likely to be independent in the transfer tasks needed for activites of daily living.

The results from this systematic review should be generalized to a wider stroke population cautiously due to the low recruitment figures for the majority of the included RCTs. Observations of lack of efficacy should also be interpreted with caution, given the uncertainty surrounding the methodological quality of the existing evidence base. Only a small number of papers with small sample sizes were able to be included in this review. Three of the selected studies recruited fewer than 20 participants [61,63,64,72], and only two recruited over 100 participants [62,65]. This could be for a number of reasons including difficulty to recruit a poststroke population to such studies. Despite the plethora of research in poststroke gait research, only 11 RCTs were selected for this review. This could be due to the difficulty of including complex interventions within an RCT design.

An RCT aims to control conditions for each arm of the study, frequently aggregating group data to provide mean values. However, no stroke is the same, recovery varies across individuals, and recovery is naturally accelerated soon after the stroke compared to those who suffered a stroke a long time ago. These factors coupled with different causes and different types of stroke, make it very difficult to control each arm of a study. Therefore, it is difficult to infer if certain interventions improve functionality post stroke or if other variables are responsible. Exploring individual change over time particularly when evaluating novel technologies with complex conditions may provide more valuable information. It has been suggested [73] that the integration of a realist evaluation perspective within an RCT design may be more appropriate and a paradigm shift for evidence-based medicine where “statistically significant benefits may be marginal in clinical practice” [74].

**Table 3:** Study, participant, and intervention characteristics and results.

Authors, year, country, study design	Number recruited (N) & final follow-up (n) overall and between groups	Gender, mean age, L/R^a^ hemisphere stroke, mean time since stroke	Intervention length/ frequency	Activity and participation outcome measure(s)	Activity and participation outcome results summary and reported *P* values
Bauer et al, 2015 [60], Austria, monocentric single-blinded RCT^b^, active leg cycling with (intervention) and without (control) FES^c^	N (n)=40 (21). 21 (12), intervention; 19 (9), control	9M/9F^d^, 64±11 years, 10R/8L, 42±45 days (control), 12M/7F, 59±14 years, 5R/14L, 62±43 days (intervention)	20 mins, 3x/week over 4 weeks. Total of 12 sessions	FAC^e^, 10MWT^f^	The intervention group increased by a median of 2 categories for the FAC and a median of 1 category for the control group (*P*=.01). No significant between-group differences found for the 10MWT (*P*=.65). Significant between-group differences found for the POMA^g^ (*P* ≤.001); however, these differences were not maintained at follow-up (*P*=.69)
Bradley et al, 1998 [61], UK, 2-arm RCT, EMG^h^ biofeedback (intervention) or EMG biofeedback with EMG switched off (control)	N (n)=23 (21); 12 (12) intervention, 11 (9) control	12M/11F, 77/68 yrs (mild/severe control), 66.6/72.4yrs (mild/se- vere intervention), 5L/16R, 35.6 days	6 weeks/ 3x/week	RMI, 10MWT, Nottingham ADL	No significant between-group differences (RMI, 10MWT, Notting- ham ADL), although all groups improved in time taken and step count for the 10MWT and all groups improved their Nottingham ADL scores
Dorsch et al, 2015 [62], USA, Phase III randomized single-blind parallel group clinical trial, participants wore accelerometers on each ankle and received speed-only feedback [67] or AF^k^	N (n)=151 (125). 73 (58) SF; 78 (67) AF	28%F/72%M **,** 65.0 ± 13.2yrs, 42%R/29%L, 8.5days [67]; 31%F/69%M, 61.8 ± 15.7yrs, 44%R/34%L, 8days (AF)	Feedback provided 3x/week, weekend use of accelerometers was optional	FAC	No significant between-group differences found for the FAC (*P*=.39), SIS^l^-16 (*P*=.68), 15-M walking speed (*P*=.96) or average daily walking time (*P*=.54)
Intiso et al, 1994 [63], Italy, 2-arm RCT, electromyographic feedback and physical therapy (intervention) or physical therapy only (control)	N (n)=16 (14), 8 (8) intervention, 8 (6) control)	9M,/7F, 53.5yrs (control), 61.3yrs (intervention), 9R/7L, 8.3 months (control), 11.3 months (intervention)	2 months/60 mins daily	BI^m^	No significant between-group differences (BI), 4/8 participants found to have significant increased BI scores
Mansfield et al, 2015 [67], Canada, single-blind RCT, accelerometer with (intervention) and without (control) feedback from physiotherapist	N (n)= 60 (57). 29 (29) intervention; 31 (28) control	20M/9F, 64yrs, 11R/16L/2B, 26 days (intervention) 16M/12F, 61.5yrs, 13R/13L/2B, 23 days (control)	3-26 days per participant in each group. Mode=11 days per participant	BBS^n^	No significant between-group differences step numbers (*P*=.39), changes in walking duration (*P*=.74), number of walking bouts (*P*=.21) or the SEQ^o^ (*P*=.48). Significant between-group differences found for daily walking activity with average cadence (*P*=.01)
Mirelman et al, 2009 [68], USA, 2-arm single-blind RCT, training with robotic device coupled with virtual reality training (intervention) or robotic device alone (control)	N (n)=18 (18), 9 (9) intervention, 9 (9) control)	15M/3F, 61yrs (control), 61.8yrs (intervention), 8R/10L, 58.2 months (control), 37.7 months (intervention)	4 weeks/60 mins 3x/week	BBS, 6MWT^p^	No significant between-group differences (6MWT), BBS results/ *P* values not reported
Salisbury et al, 2013 [64], Scotland, 2-arm feasibility RCT, routine gait re-education and orthotic device (intervention and control) with ankle foot orthosis (control) or FES (intervention)	N (n)=16 (14). 9 (8) intervention, 7 (6) control	6M/10F, 52.6yrs (control), 55.8yrs (intervention), 10R/6L, 69days (control), 51.7 days (intervention)	12 weeks/20 mins 5 days/ week	FAC, 10MWT (velocity & cadence), SIS	No significant between-group differences observed (FAC 6 weeks *P*=.53, 12 weeks *P*=.75; 10MWT velocity/cadence 6 weeks *P*=.46/ *P*=.24, 12 weeks *P*=.87; SIS 6 weeks *P*=.1, 12 weeks *P*=.3)
Shamay, 2009 [65], Hong Kong, 4-arm placebo RCT, 1. transcutaneous electrical nerve stimulation [23], 2. TENS^q^+Exercise, 3. Placebo stimulation+exercise, 4. control group (no active treatment) – home-based program	N (n)=109 (101). 29 (27) control, 28 (25) TENS, 25 (23) placebo+Ex^r^, 27 (26) TENS+Ex	85M/24F, 56.5yrs, 57.8yrs (TENS+Ex), 56.9yrs (placebo stimulation+Ex), 55.5yrs (control), 10%R/18%L [23], 10%R/17%L (TENS+Ex), 12%L/13%R (placebo stimulation+Ex), 9%L/20%R (control), 4.9yrs [23], 4.7yrs (TENS+Ex), 4.3yrs (placebo stimulation+Ex), 5yrs (control)	4 weeks/TENS: 60 mins electrical stimulation, TENS+Ex & placebo stimulation + Ex 60 mins of Ex then 60 mins electrical or placebo stimulation. Subjects attended 8 instruction sessions prior to data collection	6MWT, TUG^s^	Compared to all other groups, TENS+Ex group showed significant decreased TUG results (*P*=.01) when compared to the control and TENS group, they cov- ered more distance during the 6MWT (*P* ≤.01)
Solopova et al, 2011 [69], Russia, 2-arm RCT, conventional therapy and FES combined with progressive limb loading (intervention) or conventional therapy only (control)	N (n)=61 (61). 32 intervention, 29 control	33M/28F, 64±18yrs, 19R/42L, 9.3±4.5 days (control), 8.2±4.3 days (intervention)	2 weeks/30 mins 5 days per week	BI	No significant between-group differences, Significant improvements after the intervention in the experimental group were observed (BI *P* ≤.05)
Stein et al, 2014 [14], USA, 2-arm RCT, exercise group therapy (control) or experimental robotic therapy (intervention).	N (n)=12 (10), 12 (10) intervention, 12 (10) control)	58%M (control), 83%M (intervention), 57.6yrs (control), 56.6yrs (intervention), L/R stroke not reported, 88.5 months (control), 49.1 months (intervention)	6 weeks/60 mins 3 days per week	BBS, 6MWT, TUG, 10MWT, EFAP^t^	BBS scores favored the intervention group and the EFAP scores favored the control group. No statistically significant between-group differences observed (BBS, 6MWT, TUG, 10MWT, EFAP)
Watanabe et al, 2014 [66], Japan, 2-arm RCT single leg version of HAL^u^ (intervention) or conventional gait training (control).	N (n)=32 (22). 17 (11) intervention, 15 (11) control	11M/11F, 75.6±13.9 (control), 67.0±16.8 (intervention), 11R/11L, 50.6±33.8 days (control), 58.9±46.5 days (intervention)	4 weeks/12 20-min sessions	6MWT, FAC, TUG, SPPB^v^	No significant between-group differences were observed (6MWT, TUG, FAC, SPPB). Intervention group improved more than the control group (FAC *P*=.04)
					

^a^L/R/B: left/right/both hemisphere stroke.

^b^RCT: randomized controlled trial.

^c^FES: functional electrical stimulation.

^d^M/F: male/female.

^e^FAC: functional ambulatory category.

^f^10MWT: 10 Meter Walk Test.

^g^POMA: Performance-Oriented Mobility Assessment.

^h^EMG: electromyography.

^i^RMI: Rivermead Mobility Index.

^j^ADL: activities of daily living.

^k^AF: augmented feedback.

^l^SIS: Stroke Impact Scale.

^m^BI: Barthel Index.

^n^BBS: Berg Balance Scale.

^o^SEQ: Stroke Self-Efficacy Questionnaire.

^p^6MWT: 6 Minute Walk Test.

^q^TENS: transcutaneous electrical nerve stimulation.

^r^Ex: exercise.

^s^TUG: Timed Up and Go Test.

^t^EFAP: Emory Functional Ambulation Profile.

^u^HAL: Hybrid Assistive Limb.

^v^SPPB: Short Physical Performance Battery.

The results of the RCTs were not combined for a meta-analysis due to the varied types and quality of data collected for the primary outcome measures. It would also be difficult to compare primary outcomes across RCTs accurately as there were a wide variety of functional and participation outcome measures used across the 11 RCTs, some of which lacked validity as a measure of activity and participation.

Evidence exists supporting the need for task specificity, intensity, and repetition of motor skills in order to promote neuroplasticity and motor relearning; however, seven of the interventions in this review of RCTs were reliant on staff presence. This automatically eliminates the ability of stroke survivors to self-manage their rehabilitation, increasing both intensity and repetition within a task-specific environment.

This review included 550 participants at baseline and 474 participants at final follow-up, 260 from two studies alone [62,65]. Stroke severity can affect the rate by which individuals recover from a stroke and how they may or may not respond to interventions. Only two [61] of 11 papers in this review reported the stroke severity of their participants. Perhaps the severity was low and therefore it was difficult to infer a significant improvement of function. One paper [65] reported clinically and statistically significant results for the use of lower-limb wearable technologies with rehabilitation, although the technology was TENs, a technology that may not support a self-management paradigm and is not always tolerated by stroke survivors.

Perhaps future research should consider larger sample sizes, with valid, reliable, and responsive measurement tools ensuring clarity when reporting outcomes. Population descriptors should be used when exploring technology enhanced self-management models of poststroke rehabilitation. Outcome measures should be chosen only if they have psychometric or clinimetric properties reported. Where possible, individuals’ change over time should be captured and analyzed to ensure we begin to understand what works for whom and in what respect [75].

### Conclusion

This review found that there is little evidence in the literature to support the use of wearable technologies to improve activity and participation following a stroke. However, this review has highlighted a number of reasons for a lack of significant findings including low sample sizes, the appropriateness of RCT methodology for complex interventions, a lack of appropriate analysis of outcome data, and participant stroke severity.

## Appendix

Multimedia Appendix 1 MEDLINE search strategy.

Multimedia Appendix 2 Papers and reasons for exclusion.

Multimedia Appendix 3 Details of quality assessment.

Multimedia Appendix 4 Summary of outcome measure quality assessment.

## Abbreviations

10MWT: 10 minute walk test
6MWT: Six Minute Walk Test
BBS: Berg Balance Scale
BI: Barthel Index
EFAP: Emory Functional Ambulation Profile
EMG-BFB: Electromyographic biofeedback
FAC: Functional Ambulatory Category
FES: Functional electrical stimulation
ICF: International Classification of Functioning, Disability and Health
L/R: Left/Right hemisphere stroke
M/F: Male/Female
MCID: Minimally clinically important difference
NHS: National Health Service
MI: Motricity Index
Nottingham ADL: Nottingham Activities of Daily Living Index
POMA: Performance-Oriented Mobility Assessment
PRISMA: Preferred Reporting Items for Systematic Reviews and Meta-Analyses
RCT: Randomised controlled trial
RMI: Rivermead mobility index
SEQ: Stroke Self-Efficacy Questionnaire
SIS: Stroke Impact Scale
SPPB: Short Physical Performance Battery
TENS: Transcutaneous Electrical Nerve Stimulation
TUG: Timed Up and Go Test
