# Supplementary material for: The Effectiveness of Lower-Limb Wearable Technology for Improving Activity and Participation in Adult Stroke Survivors: A Systematic Review
Source: J Med Internet Res. 2016 Oct 7;18(10):e259. doi: 10.2196/jmir.5891 (PMC5075044; doi:10.2196/jmir.5891)
Supplement: Multimedia Appendix 1 [file jmir_v18i10e259_app1.pdf]

Multimedia Appendix 1: Medline Search Strategy

1. Stroke/ or Stroke.mp.
2. CVA.mp.
3. poststroke.mp.
4. exp Cerebrovascular Disorders/ or Cerebrovascular.mp.
5. exp Brain Ischemia/ or Brain Isch?emi$.mp.
6. exp Hemorrhage/ or exp Cerebral Hemorrhage/ or H?emorrhage.mp.
7. 1 or 2 or 3 or 4 or 5 or 6
8. Technology.mp. or exp Technology/
9. Technolog$.mp. [mp=title, abstract, original title, name of substance word, subject heading word, keyword heading word, protocol supplementary concept word, rare disease supplementary concept word, unique identifier]
10. ICT.mp.
11. IT.mp. [mp=title, abstract, original title, name of substance word, subject heading word, keyword heading word, protocol supplementary concept word, rare disease supplementary concept word, unique identifier]
12. Assistive Technology.mp. or exp Self-Help Devices/
13. Assistive Technolog$.mp. [mp=title, abstract, original title, name of substance word, subject heading word, keyword heading word, protocol supplementary concept word, rare disease supplementary concept word, unique identifier]
14. Telehealth$.mp.
15. Telecare.mp.
16. exp Rehabilitation/ or Telerehab$.mp.
17. Telemed$.mp. or exp Telemedicine/
18. 8 or 9 or 10 or 11 or 12 or 13 or 14 or 15 or 16 or 17
19. Physio$.mp.
20. Physical therap$.mp. or exp Physical Therapy Modalities/

21. Physiatric$.mp. or exp "Physical and Rehabilitation Medicine"/

22. exp Exercise/ or exp Exercise Therapy/ or exp Exercise Movement Techniques/ or Exercise.mp.

23. Biofeedback.mp.

24. Feedback.mp. or exp Feedback/ or exp Feedback, Sensory/

25. Advi$.mp.

26. Result$.mp.

27. eval$.mp.

28. observ$.mp.

29. assess$.mp.

30. Inform$.mp.

31. 19 or 20 or 21 or 22

32. 23 or 24 or 25 or 26 or 27 or 28 or 29 or 30

33. Rehab$.mp. or exp Rehabilitation/

34. Train$.mp.

35. Therap$.mp.

36. Treat$.mp.

37. Motor re-learn$.mp. or exp Motor Skills/

38. Re-educat$.mp. [mp=title, abstract, original title, name of substance word, subject heading word, keyword heading word, protocol supplementary concept word, rare disease supplementary concept word, unique identifier]

39. Re-learn$.mp.

40. Recovery enhance$.mp.

41. Promote$.mp.

42. Support$.mp.

43. Function$.mp.

44. Activit$.mp.

45. Physical$.mp.

46. 33 or 34 or 35 or 36 or 37 or 38 or 39 or 40 or 41 or 42 or 43 or 44 or 45

47. Gait$.mp. or exp Gait Disorders, Neurologic/ or exp Gait/ or exp Gait Ataxia/ or exp Gait Apraxia/

48. Ambulant$.mp.

49. exp Walking/ or Walk$.mp.

50. Locomotion$.mp. or exp Locomotion/

51. Mobil$.mp.

52. Move$.mp.

53. Motion.mp. or exp Motion/

54. 47 or 48 or 49 or 50 or 51 or 52 or 53

55. Lower Limb$.mp. or exp Lower Extremity/

56. Ambulant$.mp.

57. exp Walking/ or Walk$.mp.

58. Locomotion$.mp. or exp Locomotion/

59. Mobil$.mp.

60. Move$.mp.

61. Motion.mp. or exp Motion/

62. 55 or 56 or 57 or 58 or 59 or 60 or 61

63. 7 and 18 and 31 and 32 and 46 and 54 and 62

64. limit 63 to (english language and humans and randomized controlled trial)
