# Supplementary material for: The Effectiveness of Lower-Limb Wearable Technology for Improving Activity and Participation in Adult Stroke Survivors: A Systematic Review
Source: J Med Internet Res. 2016 Oct 7;18(10):e259. doi: 10.2196/jmir.5891 (PMC5075044; doi:10.2196/jmir.5891)
Supplement: Multimedia Appendix 2 [file jmir_v18i10e259_app2.pdf]

Multimedia Appendix 2: Papers and reasons for exclusion

Boysen (2009) not technology [58]

Dragin (2014) unobtainable [59]

Gok (2008), Tanaka (2012), Peurala (2005, 2009), Maple (2008), Tong (2006), Lau (2012), Hidler (2009), Lewek (2009), Ochi 2015, Schwartz (2009) and Van Nunen (2015) not wearable technology [32,60-70]

Peurala (2005) not RCT [71]

Kunkel (2013) feasibility study with no available data [72]

Mirelman (2010), Kakuda (2013), Paoloni (2009) and Shamay (2007), Jung (2015) doesn't assess activity and participation [54,73-76].
