# Supplementary material for: The Effectiveness of Lower-Limb Wearable Technology for Improving Activity and Participation in Adult Stroke Survivors: A Systematic Review
Source: J Med Internet Res. 2016 Oct 7;18(10):e259. doi: 10.2196/jmir.5891 (PMC5075044; doi:10.2196/jmir.5891)
Supplement: Multimedia Appendix 3 [file jmir_v18i10e259_app3.pdf]

Multimedia Appendix 3: Details of Quality Assessment

Across the included RCTs six reported that the randomisation sequence was computer generated [42,44,46-49]. Five of these were therefore judged as low risk of selection bias [42,44,46,47,49] however, the RCT by Watanabe (2014) reported that odd numbered patients were allocated to the treatment group and even numbered patients allocated to comparator. This RCT was therefore judged at high risk of bias for this domain. The remaining RCTs did not report the random sequence generation method and were judged at unclear risk for this domain. Four RCTs reported that treatment allocation was concealed [9,42,46,49] but only three of these reported an appropriate method [42,46,49]. These RCTs were therefore judged at low risk of bias for this domain. The remaining RCTs were all judged as unclear risk.

Two RCTs were reported as single-blind but did not clearly report whether participants or study personnel were blinded [47,50]. One RCT reported that both participants and therapists were not blinded [48]. One RCT reported that only the outcome assessment was blind [42]. All four of these RCTs were judged at high risk for this domain. The remaining RCTs did not report on blinding of participants and personnel. Blinding of the outcome assessment was reported by six RCTs [42,44,45,47,49], which were all judged as low risk for this domain. Two RCTs reported that the outcome assessment was not blinded and were therefore judged at high risk of bias [46,48].

Five of the included RCTs reported that either all participants completed the trial [50,51], or that numbers lost to follow-up was <20% with numbers balanced across groups and that the analysis was undertaken as intention-to-treat (ITT) [9,46,49]. These trials were judged at low risk of attrition bias. However, seven RCTs reported losses to follow up that were not accounted for in the final analysis (not ITT) [42-45,47-49]. These four RCTs were therefore judged at high risk of attrition bias (see table below for details of above).

	**Random Sequence generation**	**Allocation Concealment**	**Blinding of participants and personnel**	**Blinding of outcome assessment**	**Incomplete outcome data**	**Selective reporting**
Bauer, 2015	Low risk – computer program generated	Low risk - done by a person not involved in patient recruitment	High risk – reports that only the outcome assessment is blinded	Low risk – reported as assessor blinded	High risk – 12/21 (12%) in group one and 7/19 (35%) in group two reported as lost to follow-up. Analysed as ITT but not reported how missing values were estimated.	Low risk – reports a study protocol

Bradley, 1998	Unclear – sequence generation method not reported	Unclear – allocation process not reported	Unclear – blinding of participants and personnel not reported	Unclear – blinding of outcome assessment not reported	High risk – n lost from all groups reported and balanced across groups (~10%), but not included in final analysis (not ITT)	Unclear – indication that there is a study protocol, but unclear if the outcomes assessed are the primary outcomes in the protocol
Dorsch, 2015	Low risk - using a computer-generated simple randomisation list	Unclear – allocation process not reported	Unclear – blinding of participants and personnel not reported	Low risk - A blinded observer at each location collected data	High risk 15/73 (20.5%) in group one and 11/78 (14%) in group two not included in final analysis	Low risk – reports a study protocol
Intiso, 1994	Unclear – sequence generation method not reported	Unclear – allocation process not reported	Unclear – blinding of participants and personnel not reported	Low risk – evaluations were performed before and after the treatment by a blinded examiner	High risk 2/8 (15%) withdrew from control group and not accounted for in the final analysis (not ITT). Numbers imbalanced across groups.	Unclear – no indication that there is a study protocol and not reported what primary outcome is
Mansfield, 2015	Low risk – computer program generated	Low risk - by an investigator who was not involved in participant screening	Unclear – blinding of participants and personnel not reported	Low risk – A blinded research assistant screened and enrolled participants, conducted the assessments, processed data, and generated reports	Low risk – 3/31 (9%) withdrew from one arm but analysis undertaken by ITT	Low risk – reports a study protocol

Mirelman, 2009	Unclear – sequence generation method not reported	Unclear – allocation process not reported	High risk – reported as single-blind	Unclear – blinding of outcome assessment not reported	Low risk – reports that all participants completed the intervention	Unclear – no indication that there is a study protocol and not reported what primary outcome is
Salisbury, 2013	Low risk - using a computer-generated simple randomisation list	Low risk - consecutive numbered sealed opaque envelopes	Unclear – blinding of participants and personnel not reported	High risk – data collection was undertaken by the study physiotherapist and was non-blinded	Low risk – n lost from each group <20% and balanced across groups. Analysis by ITT	Unclear – pilot study to collect outcome measures to inform future primary and secondary outcomes
Shamay, 2009	Low risk – computer program generated	Unclear – allocation process not reported	High risk – reported as single-blind	Low risk – assessed by an assessor blinded to treatment allocation	High risk – n lost from all groups reported and balanced across groups (~10%), but not included in final analysis (not ITT)	Unclear – no indication that there is a study protocol and not reported what primary outcome is
Solopova, 2011	Unclear – sequence generation method not reported	Unclear – allocation process not reported	Unclear – blinding of participants and personnel not reported	Unclear – blinding of outcome assessment not reported	Low risk – reports that all participants completed the intervention	Unclear – no indication that there is a study protocol and not reported what primary outcome is
Stein, 2014	Unclear – sequence generation method not reported	Unclear – allocation reported as concealed but method not reported	Unclear – blinding of participants and personnel not reported	Unclear – blinding of outcome assessment not reported	Low risk – n lost from each group <20% and balanced across groups. Analysis by ITT	Low risk – no protocol reported, but both primary and secondary outcomes assessed and results presented

Watanabe, 2014	High risk – computer generated sequence but odd numbered patients allocated to treatment group and even numbered patients allocated to comparator	Unclear – allocation process not reported	High risk - participants, therapists, and the evaluator were not blinded	High risk - participants, therapists, and the evaluator were not blinded	High risk – 6/17 (35%) in the intervention and 4/15 (26.7%) were lost to follow-up and not included in the final analysis (ITT)	Low risk – no protocol reported, but both primary and secondary outcomes assessed and results presented
