# Supplementary material for: The Effectiveness of Lower-Limb Wearable Technology for Improving Activity and Participation in Adult Stroke Survivors: A Systematic Review
Source: J Med Internet Res. 2016 Oct 7;18(10):e259. doi: 10.2196/jmir.5891 (PMC5075044; doi:10.2196/jmir.5891)
Supplement: Multimedia Appendix 4 [file jmir_v18i10e259_app4.pdf]

Multimedia Appendix 4: Summary of outcome measurement quality assessment

Authors, year, country, study design	Activity and participation outcome measure(s)	Psychometric properties assessed	Clinicmetric properties assessed	Analysis method appropriate
Bauer et.al, 2015, Austria, monocentric single blinded RCT, active leg cycling with (intervention) and without (control) FES.	FAC, POMA, 10MWT	Yes	FAC and POMA estimate of MCID.	Yes
Bradley et. al, 1998, UK, two arm RCT, EMG biofeedback (intervention) or EMG biofeedback with EMG switched off (control).	RMI, 10MWT, Nottingham ADL	Yes	10 MWT unclear on MCID	Aggregated data used with ordinal scales. Rasch analysis undertaken on RMI
Dorsch et. al, 2015, USA, Phase III randomised single blind parallel group clinical trial, participants wore accelorometers on each ankle and received speed only feedback or augmented feedback.	FAC, SIS-16 average daily walking time, fastest safe 15-metre walking speed.	Yes	No	Yes for primary outcomes of average daily walking time (duration) and fastest safe 15-metre walking speed
Intiso et. al, 1994, Italy, two arm RCT, electromyographic feedback and physical therapy (intervention) or physical therapy only (control).	BI		unclear MCID, due to aggregated data	Aggregated data used with ordinal scales BI. Rasch analysis showed BI is not a unidimensional scale (de Morton 2008)
Mansfield et. al, 2015, Canada, single blind RCT, accelerometer with (intervention) and without (control) feedback from physiotherapist.	Changes in walking duration and step numbers, daily walking activity with average cadence and number of walking events (bouts), SEQ.	Not reported	MCID unclear	Yes
Mirelman, et. al, 2009, USA, two arm Single blind RCT, training with robotic device coupled with virtual reality training (intervention) or robotic device alone (control).	BBS, 6MWT	6MWT yes	6MWT unclear MCID	Aggregated data used with ordinal BBS
Salisbury, et. al, 2013, Scotland, two arm feasibility RCT, routine gait re-	FAC, 10MWT (velocity & cadence), SIS	10MWT yes	Unclear MCID for 10MWT	Aggregated data used ordinal SIS. Rasch analysis

education and orthotic device (intervention and control) with ankle foot orthosis (control) or FES (intervention).				suggests domains are unidimensional (Duncan 2003)
Shamay, 2009, Hong Kong, four arm placebo RCT, 1. transcutaneous electrical nerve stimulation, 2. TENS+Exercise, 3. Placebo stimulation+exercise, 4. control group (no active treatment) - home based programme.	6MWT, TUG	Yes	Unclear MCID for 6MWT and TUG	Yes
Solopova et. al, 2011, Russia, two arm RCT, conventional therapy and FES combined with progressive limb loading (intervention) or conventional therapy only (control).	BI		Unclear MCID due to aggregated data	Aggregated data used with ordinal scale BI. Rasch analysis showed BI is not a unidimensional scale
Stein et. al, 2014, USA, two arm RCT, exercise group therapy (control) or experimental robotic therapy (intervention).	BBS, 6MWT, TUG, 10MWT, EFAP	6MWT, 10 MWT and TUG yes. EFAP yes	Unclear MCID for all measures	Aggregated data used with ordinal scale BBS
Watanabe et. al, 2014, Japan, two arm RCT single leg version of HAL (intervention) or conventional gait training (control).	6MWT, FAC, TUG, SPPB	6MWT and TUG yes<br>FAC yes	Unclear MCID for 6MWT, TUG and SPPB	Aggregated data used with ordinal scales.
